# Evaluating the accuracy of table tennis 3D markerless motion capture based on two video cameras: a pilot study

**DOI:** 10.3389/fbioe.2026.1854164

**Published:** 2026-07-31

**Authors:** Ziwei Cao, Zikang Zhang, Yi Xiao

**Affiliations:** Shanghai University of Sport, Shanghai, China

**Keywords:** artificial intelligence, computer vision, marker-based, markerless, motion capture, openpose, table tennis

## Abstract

**Introduction:**

This pilot study presents a 3D markerless motion capture system for table tennis using binocular cameras, based on the OpenPose framework and linear triangulation to obtain 3D joint motion data. The system’s accuracy was compared to a marker-based system, and the impact of different camera.

**Methods:**

A comparative experiment was conducted between the proposed markerless system and a marker-based reference system. Three camera configurations were tested: RR-100 (right-right placement, 100 cm baseline), RR-145 (right-right placement, 145 cm baseline), and RF-100 (right-front placement, 100 cm baseline). Dynamic Time Warping (DTW) similarity of elbow joint angle curves was used as the primary accuracy metric, and statistical comparisons (p-values) were performed to identify significant differences in system errors across configurations.

**Results:**

(1) At a camera position of RR-100, the DTW similarity of the elbow joint angle curves between the markerless and marker-based systems was 0.941, 0.719, and 0.854, respectively, similar to the marker-based system. The DTW similarity reached 0.948, 0.730, and 0.887 for RR-145 and 0.949, 0.729, and 0.860 for RF-100. (2) Significant differences in system errors were found at varying baseline distances with L = RR, showing better accuracy at 145 cm (p < 0.01). (3) Significant differences in errors were found with L = RF and a 100 cm baseline, with better accuracy at RF (p < 0.01).

**Conclusion:**

(1) The markerless system, though slightly less accurate, can also capture joint motion data. (2) Increasing baseline distance improves accuracy when the camera position is fixed. (3) With a fixed baseline distance, the system with the RF camera position outperforms the RR position in accuracy.

## Introduction

Motion capture has become an important auxiliary tool in modern sports training, and its solutions can be divided into two types: marker-based and markerless (Zhou and Hu, 2008). Although the marker-based motion capture system can collect human joint motion data with high-precision, its high cost, complex operation procedures, and the interference of the wearing of marker devices on athletes’ technical movements have limited its promotion in practical applications (Colyer et al., 2018; Mündermann et al., 2006). Therefore, as the demand for real-time collection of sports data in scientific sports training continues to grow (Zhou and Hu, 2008), markerless motion capture systems have been receiving increasing attention as a solution to overcome the shortcomings of traditional marker-based motion capture systems.

In recent years, OpenPose has become an important framework for human pose assessment and motion capture (Cao et al., 2017). Recent reviews have summarized the application of such AI-based pose estimation frameworks in sports and exercise (Edriss et al., 2025). Using a multi-camera system, OpenPose is able to achieve markerless human motion capture. Currently, there have been certain studies on the application of markerless motion capture systems based on OpenPose in the field of physical movement. Zago et al. utilized a binocular camera system and the OpenPose framework to obtain 3D coordinate data of the human body, thereby verifying the feasibility of using a markerless system for gait analysis (Zago et al., 2020). Nakano et al. employed a multi-camera system and the OpenPose framework to demonstrate the feasibility of the OpenPose-based markerless system in motion capture for more complex physical movements such as countermovement jumping and pitching (Nakano et al., 2020).

Table tennis is a sport characterized by fast-paced movements, a wide variety of techniques, and rich variations. At present, the collection of table tennis motion capture data is mainly accomplished by using marker-based motion capture systems (Bańkosz and Winiarski, 2018; Zheng et al., 2021).

Zheng et al. used a marker-based motion capture system to investigate the impact of wrist joint movement on the stroke effect of table tennis players (Zheng et al., 2021). Feng et al. employed a marker-based motion capture system to collect spatial feature data of table tennis techniques and constructed a stroke action model for table tennis players (Feng et al., 2020). Bańkosz et al. used a marker-based motion capture system to explore the correlation between joint angular velocity and racket speed during forehand and backhand topspin strokes of table tennis players (Bankosz and Winiarski, 2018). However, 3D markerless motion capture for table tennis based on OpenPose remains an area that requires further research.

Therefore, the main purpose of this pilot study is to evaluate the accuracy of a 3D markerless motion capture system for table tennis, based on OpenPose framework and linear triangulation. Additionally, the impact of different camera baseline distances and positions on the measurement errors were analyzed, so as to provide practical guidance for table tennis players.

## Methods

### Participants

This study was approved by the ethics committee of Shanghai University of Sport. A total of 28 table tennis players (14 male, 14 female) were randomly recruited from the China Table Tennis College (age: 21.81 ± 1.86 years, height: 171.39 ± 9.11 cm, weight: 63.61 ± 11.04 kg, right hand shake-hands grip, skill level: Chinese national grade one; training year: 13.46 ± 2.83years). The participants were informed of the study procedures before the experiment and provided written informed consent.

### Markerless motion capture

The markerless motion capture system was implemented using a binocular camera, the OpenPose framework (version 1.7.0), and a linear triangulation algorithm. The binocular camera system was composed of two identical cameras (Sony HXR-NX80, Sony, Japan) with an image resolution of 1920 × 1080 pixels and a video capture frequency of 100 Hz. The contrast and brightness of the images were automatically adjusted using software provided by the manufacturer.

The OpenPose library (version 1.7.0) was operated in a GPU environment (GEFORCE RTX 3080, Nvidia Corporation, Santa Clara, CA, USA), independently outputting the 2D coordinates of 25 key points of the human body for each frame (Cao et al., 2017). By performing stereo calibration on the binocular camera system, the intrinsic and extrinsic parameters of the cameras were obtained. Subsequently, the linear triangulation algorithm was used to convert the 25 sets of 2D pixel coordinates obtained by OpenPose into 3D global coordinates. In this study, the three upper limb joints (right wrist, right elbow, and right shoulder) were selected for observation.

**FIGURE 1:**
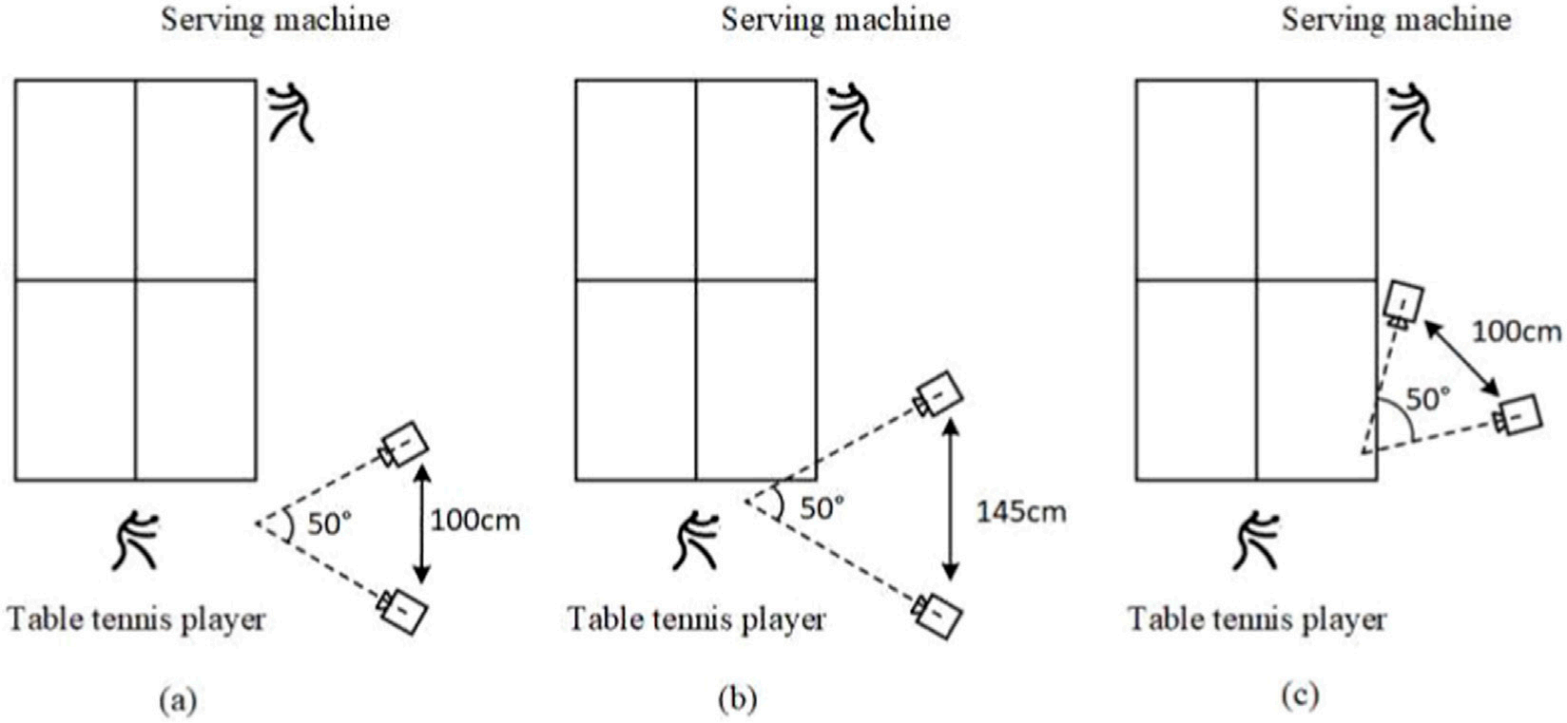
Layout Schemes of Markerless Motion Capture. **(a)** RIGHT-RIGHT-100 (RR-100): Both cameras were positioned on the right side of the athlete, with a baseline distance of 100 cm **(b)** RIGHT-RIGHT-145 (RR-145): Both cameras were positioned on the right side of the athlete, with a baseline distance of 145 cm **(c)** RIGHT-FRONT-100 (RF-100): One camera was positioned on the right side of the athlete, and the other was placed in front of the athlete, with a baseline distance of 100 cm.

To investigate the impact of different baseline distances and camera positions on the accuracy of the markerless motion capture system, three distinct binocular camera layout schemes were set up for the experiment, as shown in Figure 1.

The selection of camera configuration was based on the principle of ensuring that the target joint remains visible throughout the entire process of the athlete’s stroke. If the rays from the projection centers of the two cameras to the target joint point are parallel, the rays cannot intersect, making it impossible to compute the 3D coordinates (Hartley and Sturm, 1997; Olague and Mohr, 2002). To avoid this issue and improve the accuracy of the acquired 3D coordinate data, the angle between the two cameras was set at approximately 50°during the experiment. This ensures that the target joint was within the viewing range of both cameras while also increasing the non-parallelism of the view vectors (Olague and Mohr, 2002; Rahimian and Kearney, 2017).

### Marker-based motion capture

A marker-based high-speed infrared motion capture system (Qingtong Vision Technology Company, Shanghai, China) was employed as the reference measurement system. This system consists of 12 infrared high-speed cameras, capturing the spatial positions and joint angles of the major joints during the athlete’s stroke at a sampling frequency of 120 frames per second. The system obtains the coordinates of the body joints by detecting spherical reflective markers with a diameter of 16 mm, attached to the body surface of the athlete(Feng et al., 2020). The same three upper limb joints (right wrist, right elbow, and right shoulder) were selected for observation.

### Experimental setup

**FIGURE 2:**
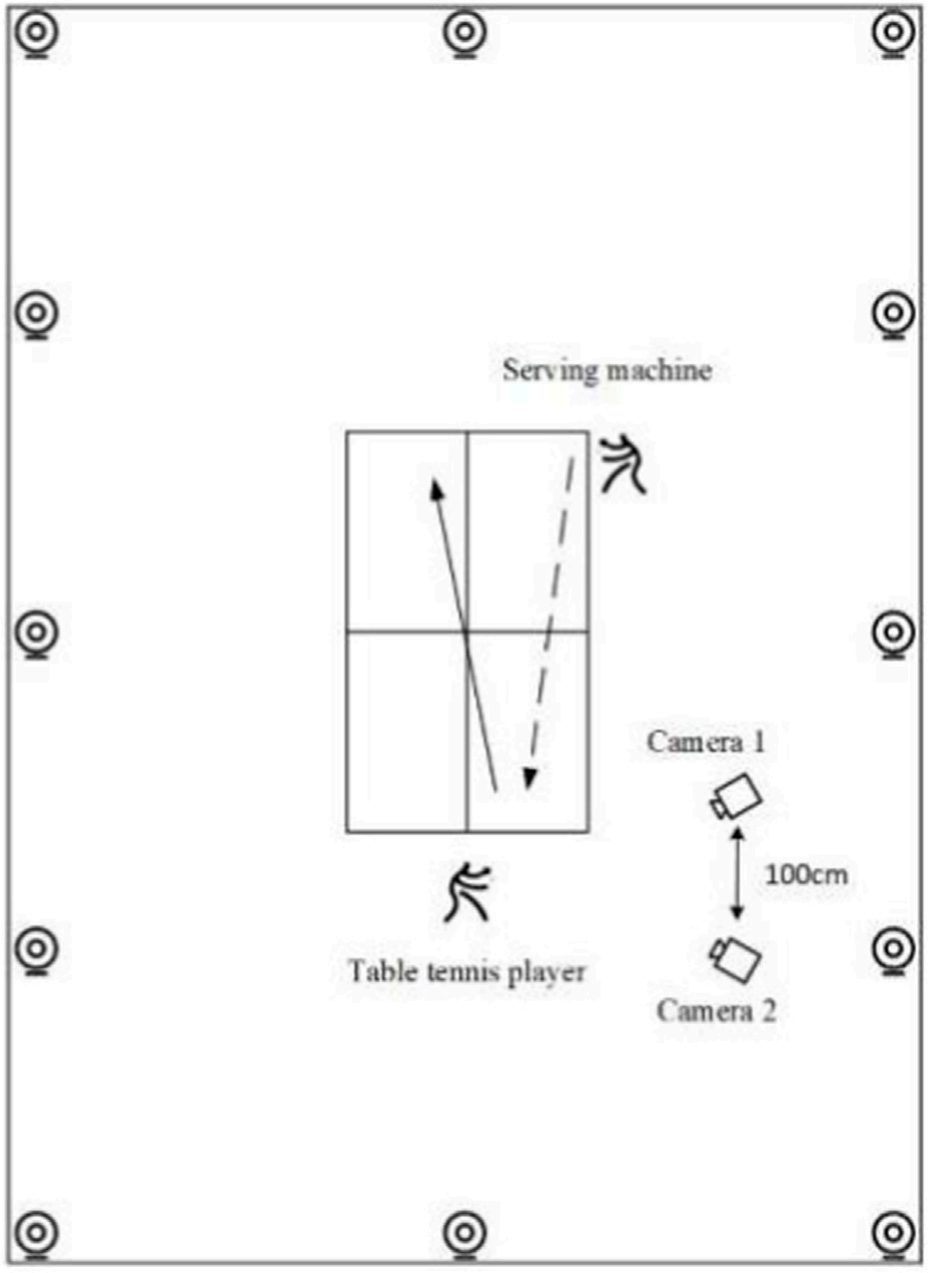
Experimental setup.

The experimental setup consisted of two parts: the marker-based high-speed infrared motion capture system and the binocular markerless motion capture system. The former included 12 infrared high-speed cameras mounted on a steel control frame on the ceiling, approximately 4 m above the ground. The binocular markerless motion capture system was placed on the ground, with the cameras positioned 1.5 m above the floor and approximately 1.5 m to the right front of the athlete, with a baseline distance of 100 cm. The experimental layout was located in the training hall on the third floor of the China Table Tennis College, as shown in Figure 2.

**FIGURE 3:**
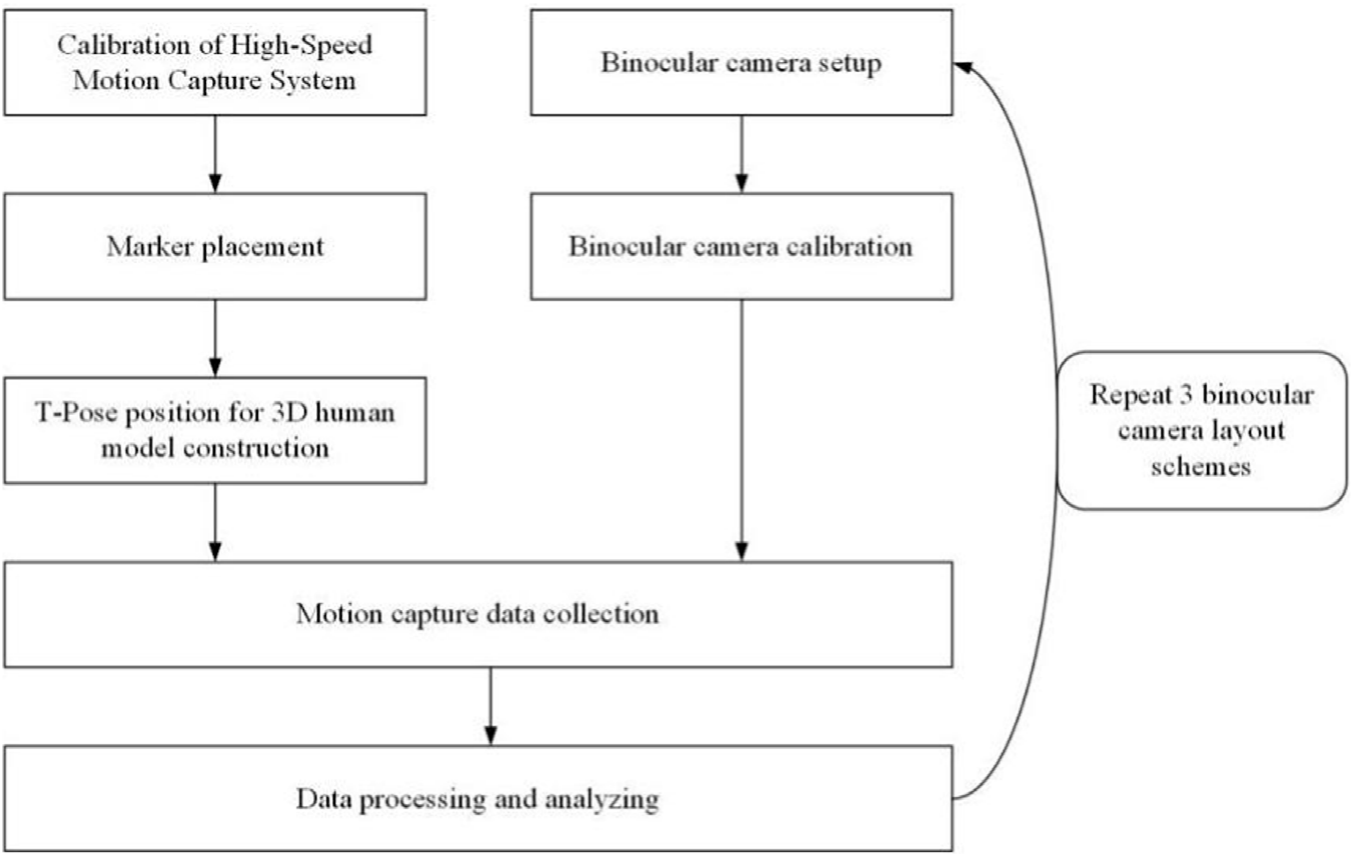
Experimental workflow.

Each participant wore a motion capture suit equipped with 23 markers(Feng et al., 2020) and used his/her forehand to strike the topspin balls five times for each camera layout. The topspin balls were served by a serving machine. During the stroke process, their joints motion data of the athlete were simultaneously collected using both the marker-based high-speed infrared motion capture system and the binocular markerless motion capture system. The specific experimental procedure was shown in Figure 3.

### 3D point detection and accuracy evaluation

**FIGURE 4:**
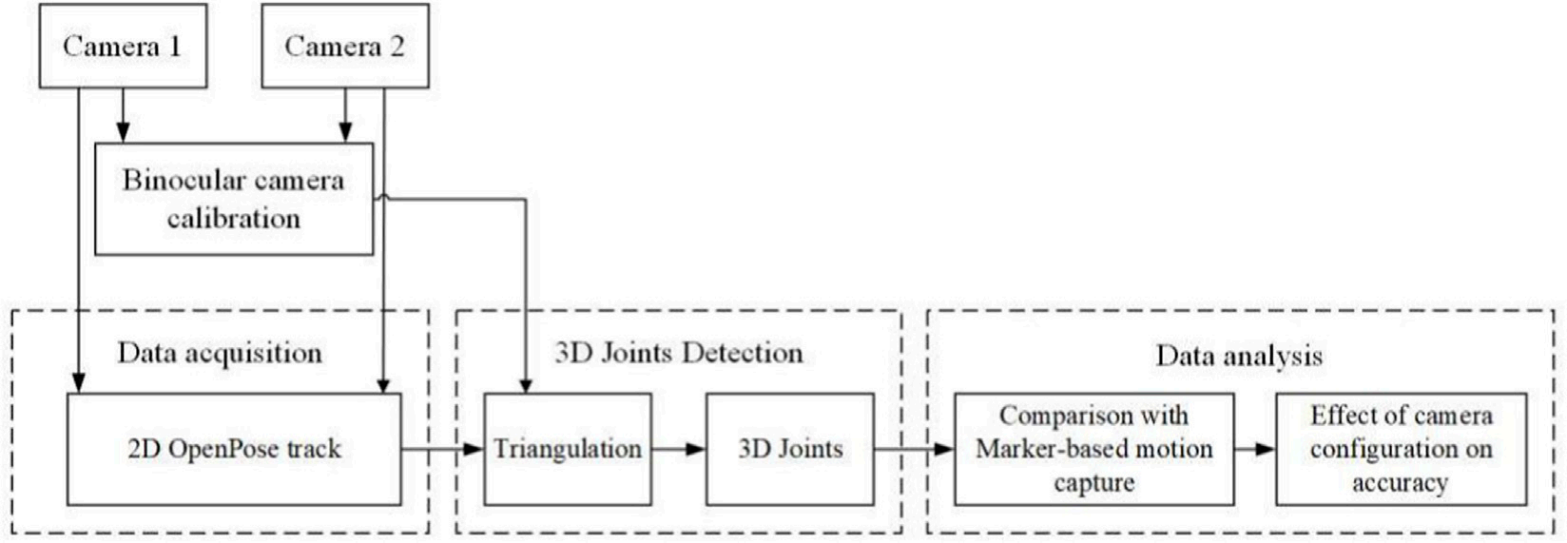
3D point detection and accuracy evaluation workflow.

The workflow for obtaining 3D coordinates of human body joints using the markerless 3D motion capture system and evaluating the accuracy of the coordinate data was shown in Figure 4. It included the following steps: binocular camera calibration, data acquisition, 3D joint detection, and data analysis.

#### Three phase division of stroke

In this study, the whole forehand stroke of table tennis players was divided into three phases: (a) backswing phase, (b) forward phase, and (c) backward phase (Zheng et al., 2021).

#### Binocular camera calibration

**FIGURE 5:**
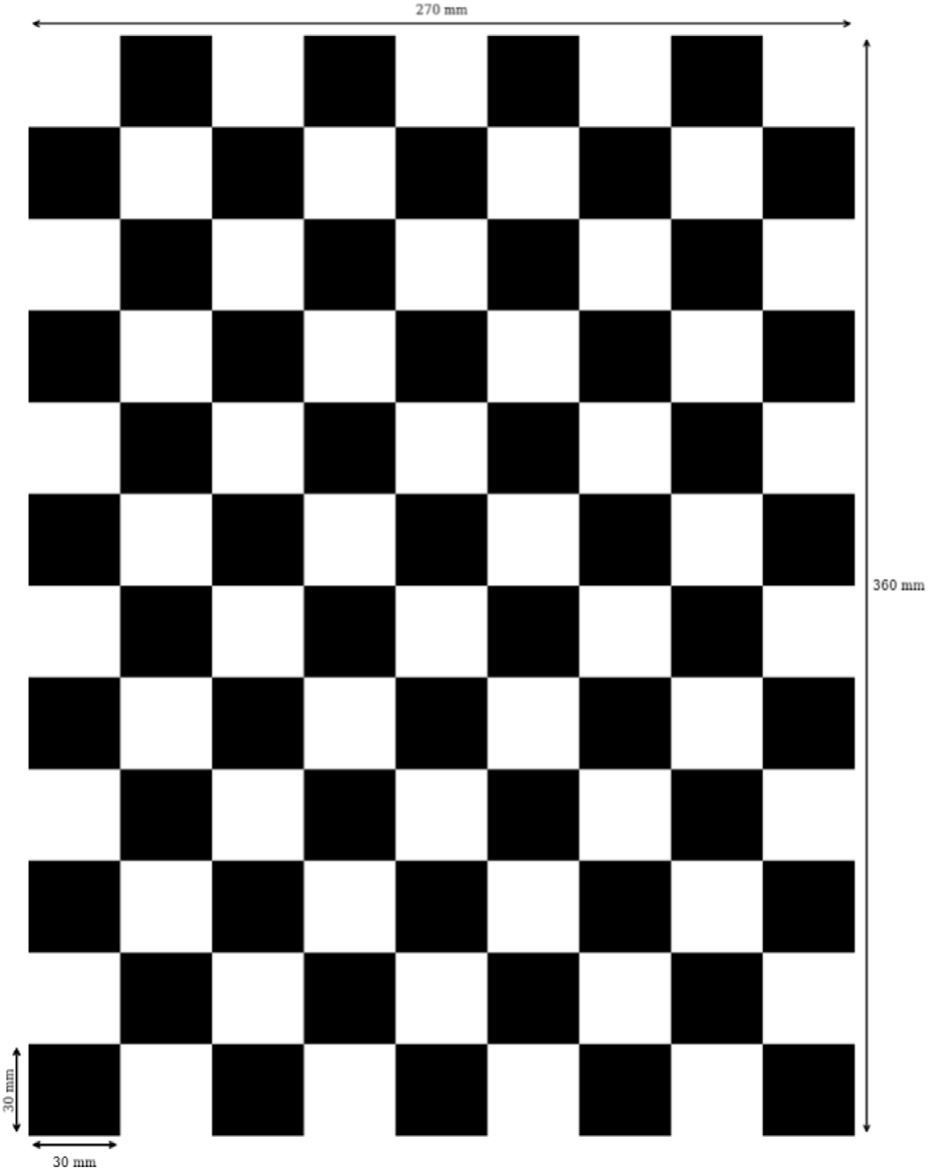
Calibration board.

The calibration of the binocular camera was performed in MATLAB (R2021b, MathWorks Inc., Natick, Massachusetts, USA) using the Stereo Camera Calibration tool (Zhang, 2002). The chessboard calibration plate used for the stereo calibration of the cameras had dimensions of 360 mm × 270 mm, with a 12 × 9 black-and-white grid array. Each square of the grid measured 30 mm in length, as shown in Figure 5.

The Stereo Camera Calibration tool returns the intrinsic parameters (intrinsic matrix and distortion coefficients) and extrinsic parameters (rotation matrix and translation vector) of the cameras. The intrinsic parameters describe the optical center and focal length of the camera, while the extrinsic parameters describe the camera’s position in the 3D scene and the relative position between the two cameras. For each of the two cameras, 50 pairs of matched calibration images were input, and image pairs with higher errors were removed and recalibrated to obtain more accurate calibration results (Zhang, 2002). To assess the calibration accuracy, the reprojection error was calculated. In this study, the calibration errors for the three different binocular camera layout schemes were all set to 0.1 pixels.

#### Data acquisition

Two videos of the athlete’s forehand stroke were captured using the binocular camera system, with one video recorded by each camera. To ensure synchronization of the data from the binocular camera system, a signal light flashing red light (twice per second) was used for video synchronization. The procedure was as follows: after both cameras were activated, turn on the signal light to ensure that both cameras can capture the flashing of the signal light. Each camera simultaneously recorded the flashing status of the signal light during the experiment. Subsequently, Adobe Premiere Pro (v2022, Adobe Systems, San Jose, CA, USA) was used to identify the frame in which each camera captured the first flash of the signal light (synchronization start frame). Using this synchronization frame as the starting point, the videos from both cameras were synchronized, and video segments with the same length capturing the athlete’s forehand stroke were extracted (synchronization end frame).

#### 3D joints detection

The intrinsic matrix, distortion coefficients, rotation matrix, and translation vector obtained from the stereo camera calibration were used for distortion correction and linear triangulation.

Firstly, the distortion coefficients were used to perform distortion correction on each frame of the captured video segment. Secondly, OpenPose was employed to processes the video segment to extract the target joint coordinates, which were saved in a txt file. The 2D coordinates returned by OpenPose are image pixel coordinates, with the origin located at the top-left corner of the image. Thirdly, using the results from the stereo camera calibration, the 2D coordinates of the target joints in each video segment were transformed into 3D coordinates through the linear triangulation algorithm. These 3D coordinates are represented in a Cartesian coordinate system relative to the camera coordinate system.

#### Segment definition and elbow joint angle calculation

The upper arm segment was defined as the vector from the right shoulder to the right elbow, and the forearm segment as the vector from the right elbow to the right wrist. The elbow joint angle was defined as the three-dimensional angle formed between these two segments at the elbow joint. The angle was calculated from the 3D coordinates of the shoulder, elbow, and wrist joints by computing the angle between the two vectors using the dot product. The angle was expressed in degree, ranging from 0° to 180°.

#### Data aggregation

For accuracy comparison, the frame-wise absolute error between the markerless and marker-based elbow joint angles was computed for the three phases of each stroke. These frame-wise absolute errors were then averaged to yield the absolute error (AE) for three phases of each stroke. Subsequently, for each participant and camera configuration, the AEs of five strokes were averaged to yield the mean absolute error (MAE) for three phases of five strokes, respectively. Paired-samples t-tests were then performed on these MAEs for each phase of all 28 participants to compare the accuracy between different camera layouts.

#### Comparison with marker-based motion capture

Due to the difference in frame rates between the markerless motion capture system and the high-speed infrared motion capture system, in order to effectively compare the data collected by the two systems, it is necessary to align their frame rates. Therefore, linear interpolation was used to resample the curves, adjusting the data from both systems to a range of {1, 101} frames (Zago et al., 2020). This study calculated the right elbow joint angles of each participant for each frame during the forehand stroke using the obtained 3D coordinates from both systems. Then, for each participant, the similarity between the elbow joint angle changing curves derived from the two systems was then calculated using the Dynamic Time Warping (DTW) algorithm(Feng et al., 2020), which is a classic time series analysis algorithm that measures the similarity between two temporal sequences.

In addition, using the marker-based motion capture system as the reference, the error analysis was performed for the markerless motion capture system during the three phases of each stroke for all 28 participants. The minimum error, maximum error, and mean error for each phase were calculated, and the standard deviation corresponding to the mean error was also provided. Furthermore, to provide a measure of accuracy of the markerless motion capture system, the Root Mean Square Error (RMSE) was further calculated to quantify the standardized absolute numerical deviation relative to the marker-based reference system. The results were presented in Table 1.

**TABLE 1:** Results of the differences between the elbow joint angles captured by the markerless and marker-based motion capture systems (degree).

Phases	RR-100				RR-145				RF-100			
	Min	Max	Mean ± SD	RMSE	Min	Max	Mean ± SD	RMSE	Min	Max	Mean ± SD	RMSE
Backswing	0.10	3.91	1.79 ± 0.51	2.06	0.05	3.45	1.14 ± 0.33	1.45	0.07	3.42	1.50 ± 0.41	1.72
Forward	2.78	13.54	7.12 ± 2.26	7.91	0.18	7.20	2.57 ± 0.75	3.33	0.26	8.64	4.50 ± 1.27	5.37
Backward	0.57	10.75	6.95 ± 2.13	7.41	0.34	7.77	3.53 ± 0.96	3.93	0.32	9.17	5.15 ± 1.47	5.64

#### Effect of camera layout on accuracy

To evaluate the impact of different baseline distances and camera placement positions on the accuracy of the markerless motion capture system, a paired-samples t-test was employed to analyze the errors between the elbow joint angles obtained from the markerless system under different camera layout schemes and those from the high-speed infrared motion capture system. Specifically, the analysis included: (1) the differences in the same camera position with varying baseline distances; and (2) the differences in the same baseline distance with varying camera positions.

## Results

### Accuracy evaluation of the markerless motion capture system

The DTW algorithm was used to calculate the similarity between the elbow joint angle changing curves during the three phases of the athlete’s stroke, obtained from the markerless and marker-based motion capture systems. The results were shown in Table 2.

**TABLE 2:** Curve similarity of elbow joint angle between different camera layouts.

Phases	RR-100	RR-145	RF-100
Backswing	0.901	0.957	0.954
Forward	0.743	0.789	0.767
Backward	0.859	0.891	0.883

When the camera position was at RR and the baseline distance was 100 cm, the similarity between the markerless system and the marker-based system for the backswing, forward, and backward phases were 0.901, 0.743, and 0.859, respectively. When the camera position was at RR and the baseline distance was 145 cm, the similarity for the backswing, forward, and backward phases were 0.957, 0.789, and 0.891, respectively. When the camera position was at RF and the baseline distance was 100 cm, the similarity for the backswing, forward, and backward phases were 0.954, 0.767, and 0.883, respectively.

According to the results of curve similarity, the markerless system exhibited higher similarity with the marker-based system during the backswing and backward phases, while the similarity was lower during the forward phase.

**FIGURE 6:**
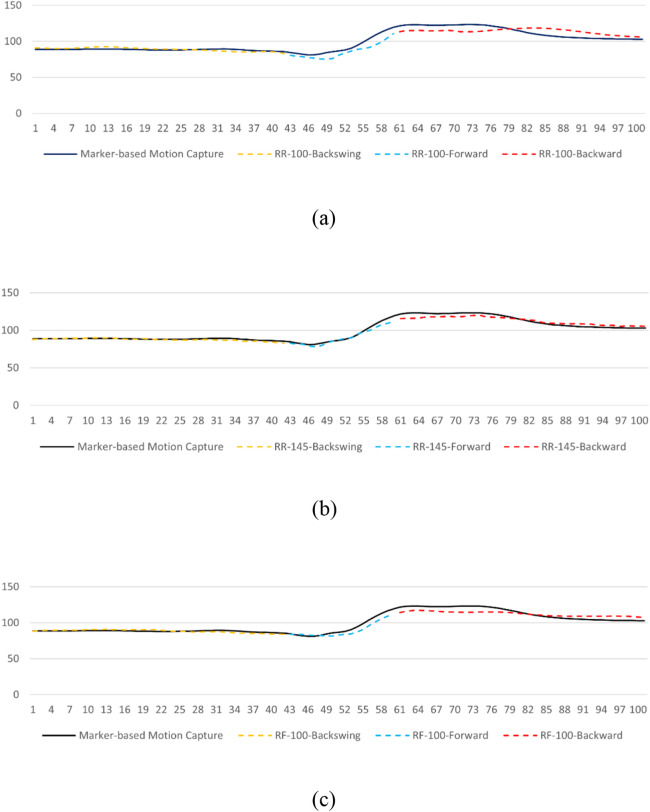
Comparison of high-speed motion capture and markerless system **(a)** Comparison of elbow joint angles between high-speed motion capture and markerless system (RR-100) **(b)** Comparison of elbow joint angles between high-speed motion capture and markerless system (RR-145) **(c)** Comparison of elbow joint angles between high-speed motion capture and markerless system (RF-100).

The comparison of the elbow joint angle changing curves captured by the markerless system and the high-speed infrared motion capture system was shown in Figures 6a–c.

The results of the differences between the elbow joint angles captured by the markerless and marker-based motion capture systems were shown in Table 1.

When the camera position was at RR and baseline distance was 100 cm, the errors of the markerless system in the backswing, forward, and backward phases were as follows: in the backswing phase, the minimum error was 0.10°, the maximum error was 3.91°, and the mean error with standard deviation was 1.79° ± 0.51°. In the forward phase, the minimum error was 2.78°, the maximum error was 13.54°, and the mean error with standard deviation was 7.12° ± 2.26°. In the backward phase, the minimum error was 0.57°, the maximum error was 10.75°, and the mean error with standard deviation was 6.95° ± 2.13°.

When the camera position was at RR and baseline distance was 145 cm, the errors of the markerless system in the backswing, forward, and backward phases were as follows: in the backswing phase, the minimum error was 0.05°, the maximum error was 3.45°, and the mean error with standard deviation was 1.14° ± 0.33°. In the forward phase, the minimum error was 0.18°, the maximum error was 7.20°, and the mean error with standard deviation was 2.57° ± 0.75°. In the backward phase, the minimum error was 0.34°, the maximum error was 7.77°, and the mean error with standard deviation was 3.53° ± 0.96°.

When the camera position was at RF and baseline distance was 100 cm, the errors of the markerless system in the backswing, forward, and backward phases were as follows: in the backswing phase, the minimum error was 0.07°, the maximum error was 3.42°, and the mean error with standard deviation was 1.50° ± 0.41°. In the forward phase, the minimum error was 0.26°, the maximum error was 8.64°, and the mean error with standard deviation was 4.50° ± 1.27°. In the backward phase, the minimum error was 0.32°, the maximum error was 9.17°, and the mean error with standard deviation was 5.15° ± 1.47°.

### Effect of different camera layout schemes on accuracy

#### Effect of camera baseline distance on accuracy

As shown in Table 3, the paired-samples t-test indicated the impact of changes in baseline distance on the systematic error during the three phases of the stroke.

**TABLE 3:** The impact of changes in baseline distance on the systematic error during the three phases of the stroke (degree).

Phases	RR-100	RR-145	*p-value*
Backswing	1.79 ± 0.51	1.14 ± 0.33	<0.001
Forward	7.12 ± 2.26	2.57 ± 0.75	<0.001
Backward	6.95 ± 2.13	3.53 ± 0.96	<0.001

When the camera position was at RR, meaning both cameras were positioned on the right side of the athlete, an increase in the baseline distance enhanced the system’s accuracy across all the three phases. Specifically, during the three phases of the stroke, the error produced by RR-100 was all significantly different from that produced by RR-145 (p < 0.001), respectively.

#### Effect of camera location on accuracy

As shown in Table 4, the paired-samples t-test indicated the impact of changes in camera position on the systematic error during the three phases of the stroke.

**TABLE 4:** The impact of changes in camera position on the system’s error during the three phases of the stroke (degree).

Phases	RR-100	RF-100	p
Backswing	1.79 ± 0.51	1.50 ± 0.41	<0.001
Forward	7.12 ± 2.26	4.50 ± 1.27	<0.001
Backward	6.95 ± 2.13	5.15 ± 1.47	<0.001

When the camera baseline distance was 100 cm, the system’s accuracy was relatively higher when the camera was positioned at RF compared to at RR. Specifically, during the three phases of the stroke, the errors generated by RR-100 were significantly different from those generated by RF-100, with all p-values less than 0.05.

## Discussion

This pilot study evaluated the accuracy of table tennis 3d markerless motion capture based on two video cameras. This system utilized the OpenPose framework and linear triangulation algorithm to collect 3D human joint motion data of table tennis players during their strokes. The accuracy of the system was evaluated by comparing the similarity of the joint movement data curves with those captured by a marker-based 3D motion capture system. Additionally, the impact of different baseline distances and camera placements on the system’s accuracy was analyzed.

The results indicated that, compared with the marker-based motion capture system, although the accuracy of the markerless table tennis motion capture system was relatively lower, it was still capable of capturing joint movement data of table tennis players and obtaining joint angle variation curves similar to those captured by the marker-based motion capture system. Error analysis showed that during the backswing phase, the errors of all the three camera layout schemes were relatively smaller, while larger errors were observed during the forward and backward phases.

The sources of error in the markerless motion capture system mainly include the following aspects:

Firstly, camera calibration errors. These errors can affect the accuracy of 3D reconstruction of human body joint movements, especially in multi-camera systems where calibration errors directly impact system precision. To address this issue, more precise calibration techniques can be employed, such as using high-precision calibration boards or multi-angle calibration methods, to reduce camera calibration errors.

Secondly, the detection accuracy of the OpenPose framework. OpenPose framework relies on joint detection from 2D images, which is limited by image resolution and the lack of depth information, leading to decreased accuracy in complex movements or rapid motions. To address this, the application of deep learning techniques can serve as a solution to enhance the robustness of the OpenPose model. Alternatively, integrating depth cameras (Liu and Chang, 2022) and additional cameras could provide richer spatial information, thereby improving detection accuracy (Huang and Nguyen, 2019; Zhang et al., 2012).

Thirdly, errors caused by data synchronization. Data synchronization is another issue that affects the accuracy of the markerless system, especially under multi-camera setups, where timing synchronization errors can lead to discrepancies in the captured data, impacting the reliability of the results. To address this issue, techniques improving hardware synchronization can be implemented to reduce errors and ensure the precision of data synchronization.

Finally, baseline distance and camera angle. The baseline distance and camera angle also have a significant impact on the accuracy of the system, especially in cases where the camera position and angle are not ideal, which may significantly increase errors. To address this issue, it is recommended to optimize the camera positions and angles during the experiment, ensuring the best viewing angles and distances to minimize errors caused by perspective deviation.

## Conclusion

This pilot study examined the feasibility of a low-cost markerless motion capture system for capturing the joint movements of table tennis players. The results indicated that: (1) the 3D markerless table tennis motion capture system can capture the elbow joint motion data of table tennis players during their stroke, however, its accuracy is relatively lower compared to the marker-based motion capture system. (2) When the camera is positioned at RR, increasing the baseline distance helps improve the accuracy of the markerless table tennis motion capture system. (3) When the camera baseline distance remains unchanged, the accuracy of the system with the camera positioned at RF is higher than that with the camera positioned at RR.

## Limitations

Although this study provides some valuable preliminary exploration into the application of low-cost markerless systems in table tennis, there are still certain limitations. First, although this pilot study recruited 28 participants for the quantitative accuracy assessment and statistical comparisons, the sample was drawn exclusively from elite players. Future research would recruit a large and diverse sample to assess the system’s validity across the target population. Second, this study examined only three camera layout schemes. Future research could explore more diverse camera setups to further investigate their impact on the system’s accuracy. Third, only the elbow joint was included in the analysis. This limits the generalizability of our findings to other joints. Future validation should include more upper- and lower-body joints. Fourth, the 3D markerless motion capture exhibited certain errors during the fast forward phase of the player’s forehand stroke. In the future, additional cameras could be added to further improve the system’s accuracy.

## Data Availability

The original contributions presented in the study are included in the article/supplementary material, further inquiries can be directed to the corresponding author.

## References

[B1] Bankosz Z. Winiarski S. (2018). Correlations between angular velocities in selected joints and velocity of table tennis racket during topspin forehand and backhand. J. Sports Sci. Med. 17, 330–338. 29769835 PMC5950751

[B2] Cao Z. Simon T. Wei S.-E. Sheikh Y. (2017). “Realtime multi-person 2d pose estimation using part affinity fields,” in Proceedings of the IEEE conference on computer vision and pattern recognition, 7291–7299.

[B3] Colyer S. L. Evans M. Cosker D. P. Salo A. I. T. (2018). A review of the evolution of vision-based motion analysis and the integration of advanced computer vision methods towards developing a markerless system. Sports Medicine-Open 4, 24. 10.1186/s40798-018-0139-y 29869300 PMC5986692

[B4] Edriss S. Romagnoli C. Caprioli L. Bonaiuto V. Padua E. Annino G. (2025). Commercial vision sensors and AI-based pose estimation frameworks for markerless motion analysis in sports and exercises: a mini review. Front. Physiology 16, 1649330. 10.3389/fphys.2025.1649330 40873758 PMC12378739

[B5] Feng Z. Xiao Y. Cao Z. W. Deng J. (2020). Evaluation of the rationality of stroke actions in youth table tennis players based on dynamic time warping algorithm: a case study of backhand topspin loop. J. Shanghai Univ. Sport.

[B6] Hartley R. I. Sturm P. (1997). Triangulation. Comput. Vision Image Understanding 68, 146–157.

[B7] Huang C.-C. Nguyen M. H. (2019). “Robust 3D skeleton tracking based on openpose and a probabilistic tracking framework,” in 2019 IEEE International Conference on Systems, Man and Cybernetics (SMC) (IEEE), 4107–4112.

[B8] Liu P. L. Chang C. C. (2022). Simple method integrating OpenPose and RGB-D camera for identifying 3D body landmark locations in various postures. Int. J. Industrial Ergonomics 91, 103354. 10.1016/j.ergon.2022.103354

[B9] MüNDERMANN L. Corazza S. Andriacchi T. P. (2006). The evolution of methods for the capture of human movement leading to markerless motion capture for biomechanical applications. J. Neuroengineering Rehabilitation 3, 1–11. 10.1186/1743-0003-3-6 16539701 PMC1513229

[B10] Nakano N. Sakura T. Ueda K. Omura L. Kimura A. Iino Y. (2020). Evaluation of 3D markerless motion capture accuracy using OpenPose with multiple video cameras. Front. Sports Active Living 2, 50. 10.3389/fspor.2020.00050 33345042 PMC7739760

[B11] Olague G. Mohr R. (2002). Optimal camera placement for accurate reconstruction. Pattern Recognition 35, 927–944. 10.1016/s0031-3203(01)00076-0

[B12] Rahimian P. Kearney J. K. (2017). Optimal camera placement for motion capture systems. Ieee Trans. Vis. Comput. Graph. 23, 1209–1221. 10.1109/TVCG.2016.2637334 27959813

[B13] Zago M. Luzzago M. Marangoni T. De Cecco M. Tarabini M. Galli M. (2020). 3D tracking of human motion using visual skeletonization and stereoscopic vision. Front. Bioeng. Biotechnol. 8, 181. 10.3389/fbioe.2020.00181 32195243 PMC7066370

[B14] Zhang Z. (2002). A flexible new technique for camera calibration. IEEE Trans. Pattern Analysis Machine Intelligence 22, 1330–1334. 10.1109/34.888718

[B15] Zhang L. Sturm J. Cremers D. Lee D. (2012). “Real-time human motion tracking using multiple depth cameras,” in 2012 IEEE/RSJ International Conference on Intelligent Robots and Systems, IEEE, 2389–2395.

[B16] Zheng C. Lu M. Zeng Y. Hu M. Geng X. Xiao Y. (2021). The impact of wrist joint movement on stroke effect during topspin forehand in table tennis. Int. J. Perform. Analysis Sport 21, 324–335. 10.1080/24748668.2021.1885839

[B17] Zhou H. Hu H. (2008). Human motion tracking for rehabilitation—A survey. Biomed. Signal Processing Control 3, 1–18. 10.1016/j.bspc.2007.09.001

